# A Long-Read Sequencing Approach for Direct Haplotype Phasing in Clinical Settings

**DOI:** 10.3390/ijms21239177

**Published:** 2020-12-01

**Authors:** Simone Maestri, Maria Giovanna Maturo, Emanuela Cosentino, Luca Marcolungo, Barbara Iadarola, Elisabetta Fortunati, Marzia Rossato, Massimo Delledonne

**Affiliations:** Department of Biotechnology, University of Verona, 37134 Verona, Italy; simone.maestri@univr.it (S.M.); maturo.mariagio@gmail.com (M.G.M.); emanuela.cosentino@univr.it (E.C.); luca.marcolungo@univr.it (L.M.); barbara.iadarola@univr.it (B.I.); elisabetta.fortunati@univr.it (E.F.); massimo.delledonne@univr.it (M.D.)

**Keywords:** nanopore sequencing, variant calling, haplotype phasing, diagnostic testing

## Abstract

The reconstruction of individual haplotypes can facilitate the interpretation of disease risks; however, high costs and technical challenges still hinder their assessment in clinical settings. Second-generation sequencing is the gold standard for variant discovery but, due to the production of short reads covering small genomic regions, allows only indirect haplotyping based on statistical methods. In contrast, third-generation methods such as the nanopore sequencing platform developed by Oxford Nanopore Technologies (ONT) generate long reads that can be used for direct haplotyping, with fewer drawbacks. However, robust standards for variant phasing in ONT-based target resequencing efforts are not yet available. In this study, we presented a streamlined proof-of-concept workflow for variant calling and phasing based on ONT data in a clinically relevant 12-kb region of the *APOE* locus, a hotspot for variants and haplotypes associated with aging-related diseases and longevity. Starting with sequencing data from simple amplicons of the target locus, we demonstrated that ONT data allow for reliable single-nucleotide variant (SNV) calling and phasing from as little as 60 reads, although the recognition of indels is less efficient. Even so, we identified the best combination of ONT read sets (600) and software (BWA/Minimap2 and HapCUT2) that enables full haplotype reconstruction when both SNVs and indels have been identified previously using a highly-accurate sequencing platform. In conclusion, we established a rapid and inexpensive workflow for variant phasing based on ONT long reads. This allowed for the analysis of multiple samples in parallel and can easily be implemented in routine clinical practice, including diagnostic testing.

## 1. Introduction

When a group of genetic variants is inherited together on a chromosome from one parent because of their genetic linkage, it defines a haplotype [1]. Reconstructing individual haplotypes, a process known as haplotype phasing or haplotyping, has important implications for the interpretation of disease risks. Multiple studies have identified haplotypes (rather than individual variants) as protective or risk factors for various conditions, such as Alzheimer’s disease [2,3,4], thrombosis [5], and breast cancer [6].

The region on chromosome 19, including the genes encoding apolipoprotein E (*APOE*), translocase of outer mitochondrial membrane 40 homolog (*TOMM40*), and apolipoprotein C1 (*APOC1*), is a hotspot for single nucleotide variants (SNVs) and haplotypes associated with Alzheimer’s disease and other age-related diseases, or with longevity [3,4,7,8,9,10]. The *APOE* ε4 haplotype (rs429358C–rs7412C) is an established risk factor for late-onset Alzheimer’s disease (LOAD) and other age-related diseases [3,4,11,12]. The risk of Alzheimer’s disease is also influenced by different variants within the *APOE* promoter when they are present in phase (in cis on the same chromosome) with the ε4 haplotype [13]. In particular, the variant –219T rs405509T is located approximately 3000 bp upstream of ε4 and significantly reduces the average age of LOAD diagnosis [4]. Many genome-wide association studies for human longevity have identified significant variants in the *TOMM40-APOE-APOC1* region [14,15,16], including a specific haplotype comprising 10 SNVs spanning 26 kb in a Chinese population [17]. However, it is currently impossible to identify carriers of such haplotypes directly by Sanger sequencing or the most common next-generation sequencing (NGS) platforms because they cannot generate reads longer than ~1500 bp from a single DNA molecule. Methods that can directly report haplotype phasing in this genomic region, and in others associated with disease risks, may allow earlier diagnosis and more effective therapy to limit the disease burden.

Technical issues and high costs currently hinder the assessment of haplotypes in clinical settings. Haplotype phasing traditionally involves the analysis of multiple individuals from the same family, to indicate the chromosomal origin of each variant [18]. However, pedigree-based haplotype inference depends on the availability of family DNA samples and cannot phase de novo variations that have arisen in the last generation. Long-range PCR and fluorescence in situ hybridization allow the direct haplotyping of particular homozygous loci, but are not useful for genome-wide analysis [19]. Second-generation NGS technologies, such as Illumina sequencing by synthesis, produce short reads (up to 300 bp) that usually span no more than a single variant, and thus allow only indirect haplotype reconstruction based on statistical inferences from population genotyping data. [18]. To overcome these issues, several library preparation methods that preserve haplotype information in short-read sequencing data have been proposed. These include mate-pair libraries, the single-cell DNA template strand sequencing (Strand-seq) [20], the proximity-ligation libraries of the Chicago [21], and Chromosome Conformation Capture (Hi-C) [22]. More recently, the company 10× Genomics launched a novel microfluidics-based library preparation approach generating linked reads that can be assembled into long haplotypes [23]. Although these approaches allow for accurate haplotyping, ad hoc library preparation protocols require additional sample preparation time and attract higher costs. 

Third-generation sequencing technologies developed by companies such as Oxford Nanopore Technologies (ONT) and Pacific Biosciences (PacBio) can facilitate rapid and direct haplotyping due to the production of long sequencing reads (>10 kb) [18,24,25,26]. Differently to short-read sequencing platforms, which rely on amplification to enlarge clusters of a given DNA template, third-generation sequencing platforms directly analyze single DNA molecules in real-time. In particular, the ONT platform identifies DNA bases by measuring the changes in electrical conductivity generated while DNA strands pass through a biological pore [27]. However, ONT technology has a higher error rate than other NGS methods (~10%) and specific adjustments, namely bioinformatic pipelines that deal with this drawback, are therefore required to fully exploit the opportunities provided by ONT sequencing [28]. Some phasing tools that enable haplotype phasing based on ONT sequencing have been developed [29,30,31], but performance benchmarking analysis has yet to be published and there is no standard pipeline for this type of analysis. Moreover, the impact of sequencing coverage on the accuracy of reconstructed haplotypes is unclear. 

To address the lack of guidelines for users wishing to perform haplotype phasing based on ONT sequencing reads, we presented a workflow for SNV calling and haplotype phasing of variants localized in a region of interest. The method exploits available and validated software and requires only a modest number of ONT reads, thus enabling efficient haplotype phasing at low costs in clinical settings.

## 2. Results

### 2.1. Optimization of APOE Locus Amplification

**Figure 1:**
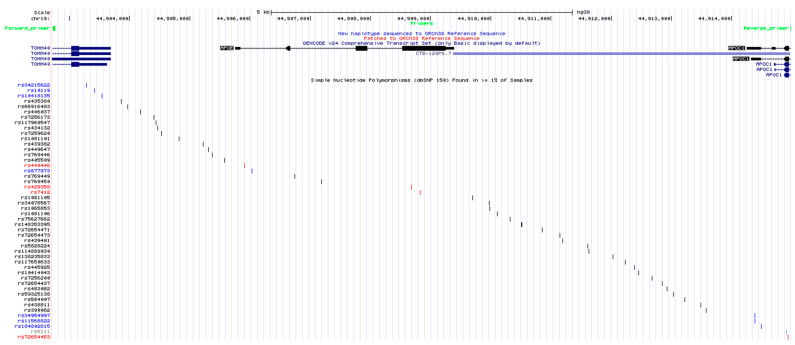
Target region selected for this the study. The genomic region chr19:44,902,730–44,915,006 (hg38) visualized in the UCSC Genome Browser, identifying the single-nucleotide polymorphisms (SNVs) included in dbSNP. SNVs indicated with gray ticks represent synonymous variants in coding regions. SNVs indicated with red ticks represent non-synonymous variants in coding regions. SNVs indicated with blue ticks represent variants in untranslated regions. SNVs indicated with black ticks represent either variants in intronic regions or upstream/downstream gene variants. The positions of the PCR primers are shown in green.

The genomic region chr19:44,902,730–44,915,006, comprising the whole *APOE* gene as well as its promoter and small parts of *TOMM40* and *APOC1*, was selected as a target region because it includes several SNVs and haplotypes previously linked to Alzheimer’s disease and other age-related diseases (Figure 1 and Supplementary File S1). According to Illumina whole-genome sequencing data and the GIAB dataset, five available samples showed numerous heterozygous SNVs within this genomic locus (Table 1) and were thus used as case studies for the analysis of variant calling and haplotype phasing in the target region.

**Figure 2:**
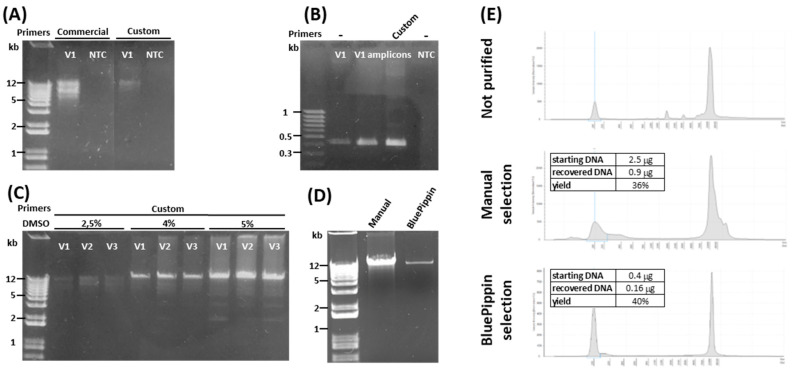
Amplification of the *APOE* locus. (**A**) Agarose gel electrophoresis of amplicons generated from sample V1 genomic DNA using LongAmp Taq Polymerase (NEB) and commercial primers (Qiagen) or custom primers. The expected amplicon size is ~12 kb. (**B**) Agarose gel electrophoresis of amplicons generated from sample V1 genomic DNA or from PCR products (V1 amplicons) generated in (**A**), using nested primers designed to amplify a 412-bp product from *APOE* exon 3. (**C**) Agarose gel electrophoresis of long-range PCR amplicons generated using custom primers in the presence of 2.5%, 4%, or 5% DMSO. (**D**) Agarose-gel electrophoresis of amplicons generated using custom primers and 4% DMSO and further selected by gel excision and on-column purification (manual) or the BluePippin device. (**E**) Capillary electrophoresis of the same products from (**D**). Starting and recovered μg refer to the amount of amplicon before and after size selection and the relative yield. NTC = no-template control.

To amplify this ~12-kb genomic region, a long-range PCR protocol was tested using either commercially available or custom-designed primer pairs (Figure 2A). Both primer pairs produced amplicons >10 kb that included the target locus, as demonstrated by the positive amplification of *APOE* exon 3 by nested PCR (Figure 2B). However, the commercial primers produced multiple amplicons including nonspecific ones with a molecular weight <12 kb, whereas the custom primers generated a cleaner but consistently weaker product (Figure 2A). To increase the amplification efficiency of the custom primers, allowing the production of enough DNA for downstream analysis, the reactions were supplemented with different amounts of DMSO and tested on three distinct biological samples. At 4% DMSO, the custom primers achieved the best compromise between efficiency and specificity (Figure 2C), despite yielding minor nonspecific products with lower molecular weights than expected (Figure 2C,E). Therefore, we used two different approaches to purify the specific 12-kb PCR products: Manual gel excision followed by on-column purification, and automatic selection using the BluePippin device. Both methods purified similar amounts of DNA (36% and 39%, respectively) but the BluePippin method yielded a significantly cleaner product, verified by manual and automatic electrophoretic analysis (Figure 2D,E). The *TOMM40-APOE-APOC1* target region was therefore amplified in all samples using the custom primers in the presence of 4% DMSO with subsequent BluePippin selection.

### 2.2. Variant Calling Performance Using ONT Data

The five samples with available genotyping data (Supplementary File S1 and Table 2) were amplified in the target locus, multiplexed, and sequenced on a R9.4 flow-cell, yielding an average of 47,835 reads per sample (Table 3). An average of 99.99% MinION PASS reads mapped properly to the target region and 98.07% of these reads spanned the entire 12-kb target.

**Figure 3:**
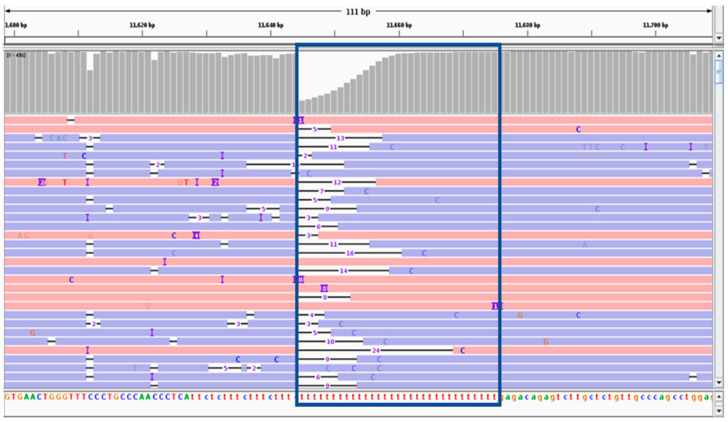
**Integrative Genomics Viewer** (IGV) visualization of ONT mapped reads from sample V1 at position 11,658. IGV visualization showing a region where a homozygous SNV at position 11,658 was not identified based on ONT data. The blue box shows that the SNV occurs within a long homopolymer run.

**Figure 4:**
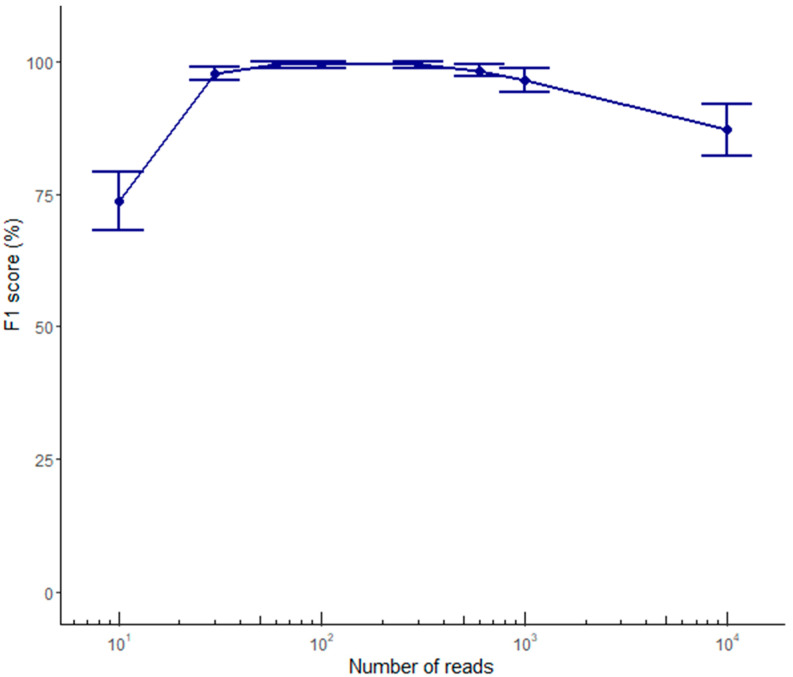
Accuracy of SNV calling from ONT reads. The graph shows the harmonic mean of precision and recall for SNV calling as an F1 score averaged across samples, when different numbers of ONT reads (10, 30, 60, 100, 300, 600, 1000, or 10,000) are used. Error bars represent the standard error.

Variant calling was carried out using sets composed of 10–10,000 ONT reads and we compared the results with the set of ground-truth variants derived from Illumina data. ONT-based variant calling showed poor results for indels, regardless of the sequence coverage, generating an average number of true positive (TP), false negative (FN), and false positive (FP) calls of 0.2, 1.4, and 2.2, respectively, at 60× coverage, and no improvement at greater sequencing depth. In contrast, SNV calling was already highly accurate when using 60 reads, generating an average number of TP, FN, and FP calls of 9.8, 0.2, and 0.0, respectively (Table 4). Only one homozygous SNV from sample V1 (found within a homopolymer run) was not properly called (Figure 3). This demonstrates that variant calling based on ONT reads is reliable only for SNVs, as long as they are not embedded in long homopolymer runs. Interestingly, the SNV calling performance reached a plateau from 60× to 300× coverage, and started to decline at 600× coverage, as shown by the decrease in the F1 score (Figure 4)

### 2.3. Optimization of a Haplotype Phasing Pipeline Based on ONT Data

The reference haplotypes for three of the five samples (V1, V2, and V3) were identified using the 10× Genomics Chromium platform by whole-genome sequencing at an average coverage of 33× (Supplementary File S2 and S3). The 10× Genomics analysis provided phasing information for 39 of the 40 heterozygous variants identified across these samples (Table 2). For the two remaining samples, haplotypes were determined based on trio analysis, starting with the publicly available VCF file of individuals from the 1000 Genomes Project (Table 2 and Supplementary File S3).

**Figure 5:**
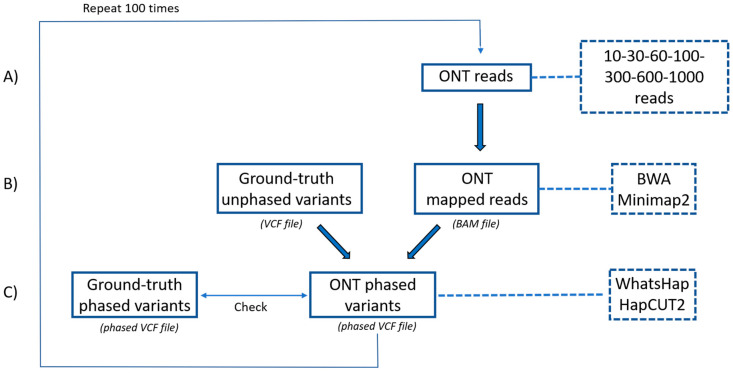
Experimental pipeline to assess the performance of alignment and phasing software. (**A**) Sets ranging from 10 to 10,000 ONT reads were considered. (**B**) Variants in the region of interest were identified using the highly accurate Illumina platform and stored in a VCF file (ground-truth unphased variants). ONT reads were mapped to the reference sequence using either BWA or Minimap2, producing a BAM file. (**C**) VCF and BAM files constitute the input files for the phasing software (WhatsHap or HapCUT2), which generated a phased VCF file allowing haplotype reconstruction. The ONT-phased VCF file was then compared to a reference-phased VCF file (ground-truth phased variants). Analysis was carried out 100 times to obtain more robust estimates of accuracy.

**Figure 6:**
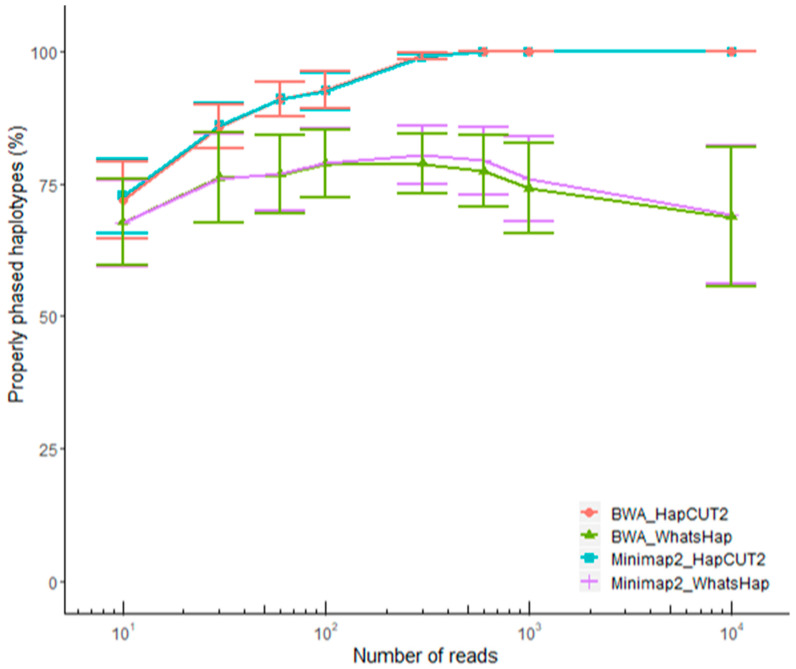
Accuracy of full haplotype reconstruction. Haplotypes were called 100 times using all possible combinations of two alignment and phasing software and different read numbers (10, 30, 60, 100, 300, 600, 1000, and 10,000). The graph shows the frequency at which the fully correct haplotype was called (averaged across samples) with error bars representing the standard error.

Compared to reference haplotypes, SNVs called at 60× coverage based on ONT data were all correctly phased by the variant calling pipeline, which used Minimap2 for alignment and WhatsHap for phasing (Table 2). In contrast, we could not evaluate indel phasing because this type of variant was not properly called by ONT. In order to assess the capability of ONT data to phase both SNVs and indels, the set of SNVs properly called by ONT (all true positives) was integrated with the truth set of indels (derived from reference VCF data) and phased solely using ONT data. At this aim, we assessed the performance of different software combinations with different numbers of ONT reads. In particular, we compared the haplotypes obtained when using Minimap2 [32] or BWA mem [33] for alignment, HapCUT2 [29] or WhatsHap [30] for phasing, and read sets ranging from 10 to 10,000 (Figure 5). In all cases, accuracy was estimated over 100 iterations. When starting from 600 read groups, all software combinations using HapCUT2 for phasing identified the correct haplotype with 100% accuracy for all samples, and the alignment software had a minimal impact on phasing accuracy (Figure 6). These results demonstrated that phasing both SNVs and indels is possible with no residual errors when exploiting at least 600 ONT reads using the combination of Minimap2 or BWA with HapCUT2.

## 3. Discussion

Despite the importance of haplotype reconstruction for the interpretation of disease risks and therapeutic responses, haplotype assessment in clinical settings is hindered by technical difficulties and high costs. Short-read sequencing (e.g., the Illumina platform) is regarded as the state-of-the-art approach for SNV discovery in the clinic. However, direct haplotyping is restricted to ~600 bp due to the maximum read length; thus, haplotypes in longer regions can only be inferred by statistical analysis. Emerging approaches such as the Hi-C method or 10× Genomics linked-read sequencing provide additional opportunities for haplotype analysis, but they require laborious and costly sample preparation methods and/or expensive equipment. Moreover, 10× Genomics announced the discontinuation of its chromium genome and exome product lines at the end of June 2020 [34].

Third-generation sequencing methods such as the ONT platform offer a promising alternative for direct haplotyping thanks to the production of long reads. Although its per-base accuracy and cost per base have improved significantly, ONT is not yet on par with short-read platforms [35,36,37]. However, in contrast to other long-read technologies such as PacBio SMRT sequencing, ONT provides flexibility in terms of data acquisition because the user can stop the sequencing run having reached the desired throughput (real-time data analysis). Moreover, the ONT platform is based on affordable instruments such as the MinION sequencer ($US 1000) and it can exploit sequencing flow cells with different output capacities, allowing a wide range of applications. ONT sequencing may therefore represent a cost-effective solution for direct SNV calling and phasing or may complement short-read technologies for the phasing of variants previously identified using these platforms.

One drawback of ONT sequencing for variant phasing is the absence of a well-defined and easy-to-use workflow from sample preparation to ONT data analysis. We therefore established a simple laboratory workflow and bioinformatics pipeline to call and phase heterozygous SNVs in any region of interest based on sole ONT sequencing. In addition, we showed that ONT reads can also be used to reconstruct correct haplotypes for both SNVs and indels, when the latter have been previously identified by high-accuracy methods such as Illumina or Sanger. The work identified the optimal coverage and the best combination of software required for variant phasing based on ONT reads, aspects not dissected in the available literature yet. Compared to currently available approaches, our workflow produces reliable results in a short time and with minimal additional costs, due to the straightforward multiplexing of samples in a single flow cell.

Attempts of using the ONT platform for direct haplotype phasing have been reported since its release to the community through the MinION Access Program (MAP). For example, Ammar et al. [38] generated nanopore reads to detect variants and haplotypes in genes involved in drug responses, whereas Stancu et al. [39] used whole-genome nanopore sequencing reads to phase heterozygous variants previously identified using the Illumina platform in a trio-based analysis. Leija-Salazar [40] evaluated the ability of nanopore sequencing to phase variants in the *GBA* gene in order to identify patients suffering from Gaucher’s disease or Parkinson’s disease. However, these feasibility studies did not determine the accuracy of haplotyping. Recently, the performance of four SNV-calling tools was evaluated on nanopore data, but the authors did not consider the impact of phasing tools [35].

We addressed the lack of benchmarking data by assessing the impact of alignment and phasing tools on variant calling and phasing accuracy with reference to sequence coverage. By calculating the percentage of properly reconstructed haplotypes, we showed that the phasing software has a much greater impact than the alignment software. Specifically, the medaka_variant workflow provided by ONT (integrating Medaka, Minimap2, and WhatsHap) called SNVs with high accuracy in all samples, with the exception of one SNV embedded in a homopolymer run. Optimal performance was achieved using 60–600 ONT reads. Interestingly, the SNV calling accuracy declined significantly when we used 1000 and 10,000 reads, which is important because high sequence coverage is very common in the context of target resequencing studies [41,42,43]. Users of the Medaka tool should pay particular attention to this drawback. In contrast, the variant calling pipeline based on sole ONT data performed less well for the identification of indels. This is likely to reflect the known bias of ONT reads, which are especially prone to errors in homopolymer runs [26,41,44]. Improvements in base-calling accuracy are constantly contributing to reduce this type of errors, with Bonito now representing a very promising base-caller [45,46]. Moreover, the R10.3 chemistry recently released by ONT is expected to improve the accuracy of indel calling because it uses pores with a longer barrel and a dual reader head, which achieves better raw read accuracy even in homopolymer regions [44,47]. Although indel calling from R9.4 data is not yet accurate, we showed that ONT reads can be used to accurately phase both SNVs and indels when these have already been identified using a more accurate sequencing approach (such as Illumina or Sanger). For this purpose, we have established a second-step pipeline where ONT reads are aligned again to the reference genome using BWA or Minimap2 and phasing of variants from a given VCF file is defined with HapCUT2. For this phasing step, 600 ONT reads allowed the recovery of fully correct haplotypes.

Our proof-of-concept study focused on the *APOE* locus, a clinically relevant genomic region, allowing for the analysis of numerous SNVs and haplotypes that have been linked to Alzheimer’s disease and other age-related diseases. For example, the proposed workflow may be used to assess the phasing of SNVs >3000 bp upstream of the *APOE* gene that may affect the disease outcome when present in the cis configuration [17]. Moreover, because SNV frequencies within the *APOE* locus are highly skewed to population-specific haplotypes [12], our workflow will allow wider population studies to assess how the ancestral background within different sites of the *APOE* gene shapes the disease phenotype. Our workflow can also be modified easily for the analysis of other genomic regions containing clinically relevant haplotypes. The enrichment of the *APOE* target locus was achieved by direct amplification of the selected region using long-range PCR, and this approach could be applied to any target of interest. Simple experimental parameters (i.e., PCR primer position and buffer composition) were tuned to maximize amplicon production and both tested methods for amplicon purification yielded enough DNA for ONT sequencing. Even so, the generation of long amplicons can be challenging in repetitive/highly homologous or GC-rich genomic targets. Innovative target-enrichment approaches such as the CRISPR/Cas9-based approach [35,48] and X-Drop technology [49], recently coupled to nanopore sequencing, may help to overcome the limitations of a PCR-based workflow and thus facilitate the broader implementation of our bioinformatics pipeline.

Given that the established pipeline requires only 600 reads per sample, multiplexing to 96 samples using the Native Barcoding Kit can be achieved in a single MinION flow cell, reducing costs to ~€20 per sample. Costs could be reduced even further by using the recent ONT Flongle flow cells, which are specifically developed for applications requiring rapid, low-cost analysis such as diagnostic applications. The simplicity and versatility of our proposed workflow therefore makes it ideal for clinical settings, where haplotype information could facilitate decision-making processes related to disease management [50].

## 4. Materials and Methods

### 4.1. DNA Extraction

DNA of donors V1, V2, and V3 was extracted from 2 mL of whole blood using the Invisorb Mini Kit (Stratec Molecular, Berlin, Germany) following the manufacturer’s instructions. DNA purity was assessed using a Nanodrop 1000 spectrophotometer (Thermo Fisher Scientific, Waltham, MA, USA) and DNA integrity was assessed by capillary electrophoresis using a TapeStation 2200 system (Agilent Technologies, Santa Clara, CA, USA). DNA was quantified using the Qubit dsDNA HS Assay kit (Thermo Fisher Scientific).

### 4.2. PCR Amplification of The Target Region

Long-range PCR was carried out in a 25-µl reaction containing 100 ng of genomic template DNA, 0%, 2.5%, 4%, or 5% DMSO, LongAmp Taq Polymerase 2× master mix (New England Biolabs, Ipswich, MA, USA), and 0.4 µM of one of the following primer pairs: Qiagen forward primer (Hs_APOE_01_LR 5′-CCG TCC ACT TTC CCA TCT CCT CGG TAT AAA TC-3′) and Qiagen reverse primer (St000357847 5′-AGA GTC AGG AAG ATT GAG AGG TGA GAG TGC-3′), or custom forward primer (5′-CTT CAC GGG ACT CAG TGA GAG GAA CAG ATT C-3′) and custom reverse primer (5′-TAG CCA CCT TCA CTC TTC CAA ATC TCC AA-3′). The reaction was heated to 94 °C for 3 min, followed by 35 cycles of 94 °C for 30 s, 64 °C for 60 s, and 65 °C for 9 min 20 s, and a final extension at 65 °C for 10 min. PCR products generated using the custom primers were separated by agarose gel electrophoresis and purified using the Zymoclean Large Fragment DNA Recovery Kit after manually excising the gel band (Zymo Research, Irvine, CA, USA). Alternatively, we applied the high pass protocol for fragments larger than 10 kb on BluePippin (Sage Science, Beverly, MA, USA) using a 0.75% agarose gel cassette. The appropriate size of purified amplicons was verified by capillary electrophoresis on the TapeStation 2200 system. An average of four reactions per sample was necessary to yield enough amplified DNA for nanopore sequencing.

### 4.3. 10× Genomics Library Preparation, Sequencing, and Data Analysis

Whole-genome sequencing libraries were constructed using the Chromium Gel Bead and Library Kit (10× Genomics, Pleasanton, CA, USA) with the Chromium instrument (10× Genomics) at the Functional Genomics Center of Zurich. Sequencing was performed at Macrogen (Seoul, Korea) on an Illumina (San Diego, CA, USA) Hiseq X producing 400 million paired-end reads of 150 p. LongRanger v2.2.2 was used to map Illumina reads to the hg38 human reference genome, and to call and phase the variants. Only variants with the “PASS” flag were retained for subsequent analysis. Unphased and phased heterozygous variants present in the target region were stored in two separate VCF files. The VCF files of the reference samples NA12878 and NA12892 were downloaded from ftp://ftp-trace.ncbi.nih.gov/1000genomes/ftp/technical/working/20140625_high_coverage_trios_broad/CEU.wgs.consensus.20131118.snps_indels.high_coverage_pcr_free_v2.genotypes.vcf.gz. Variants in the region of interest were extracted from the VCF files and genomic coordinates were lifted from the hg19 to the hg38 reference genome using CrossMap v0.3.0 [51]. The phasing of selected variants was determined using GATK PhaseByTransmission v3.8.1 [52].

### 4.4. ONT Sequencing and Data Analysis

ONT MinION 1D libraries were prepared from purified amplicons (~1 μg for each sample) using the Native Barcoding Genomic DNA Kit (SQK-LSK108) in accordance with the manufacturer’s instructions (Oxford Nanopore Technologies, Oxford, UK). The five samples were sequenced on a single R9.4 flow-cell in a 48-h sequencing run using the NC_48Hr_sequencing_Run_FLO_MIN106_SQK-LSK108 protocol and MinKNOW v1.2.8. Base-calling of raw fast5 files was carried out using Guppy v3.6.0 with high-accuracy mode. Reads were then demultiplexed using Guppy v3.6.0 by requiring the presence of barcodes at both ends of the read (--require_barcode_both_ends). Reads with quality <7 and length <11,276 nt or >13,276 nt were discarded using NanoFilt [53]. Reads were randomly subsampled using seqtk sample v1.3-r106 (https://github.com/lh3/seqtk). Variants were called using the medaka_variant subroutine of Medaka v0.13.0 (https://github.com/nanoporetech/medaka) after mapping ONT reads with Minimap2 v2.17-r941 [32] to hg38 region chr19:44,899,031–chr19:44,916,122, which we describe hereafter as the hg38_chr19_APOE_locus. Only variants overlapping hg38_chr19_APOE_locus:3730–15948 and with a “PASS” flag were retained using bedtools intersect [54]. Variant calling performance was evaluated using hap.py script (https://github.com/Illumina/hap.py).

Haplotyping was performed by mapping ONT reads to the hg38_chr19_APOE_locus using Minimap2 v2.17-r941 [32] or BWA mem v0.7.17-r1188 [33] for alignment to produce BAM files. HapCUT2 v1.3.2 [29] or WhatsHap v1.0 [30] were used to phase the heterozygous variants stored in the Illumina VCF files, relying on the information included in the BAM file. The accuracy of haplotype phasing was determined by counting the number of times that the entirely correct haplotype was reconstructed for each sample, over 100 iterations on different sizes of read sets, ranging from 10 to 10,000 reads. All the scripts are available at https://github.com/MaestSi/ONT_preprocessing and https://github.com/MaestSi/ONToHap.

## 5. Conclusions

The work presented provides broadly applicable guidelines, namely number of reads and bioinformatic software, to exploit ONT data for the phasing of genetic variants in a selected region of interest. The rapid and inexpensive workflow proposed in this work allows for the analysis of multiple samples in parallel and can easily be implemented in routine clinical practice, including diagnostic testing.

## Figures and Tables

**Table 1:** Genetic variants in the region of interest. The columns show the identification code in dbSNP (dbSNP ID), the hg38-based coordinate positions (Pos.), and the reference (Ref.) and alternative (Alt.) nucleotides of each variant.

dbSNP ID	Pos.	Ref.	Alt.
rs893292251	chr19:44902764	C	T
rs34215622	chr19:44903281	C	CG
-	chr19:44903416	G	A
-	chr19:44904398	C	T
rs7259620	chr19:44904531	G	A,C
rs449647	chr19:44905307	A	T
rs769446	chr19:44905371	T	C
rs405509	chr19:44905579	T	G
-	chr19:44905832	C	T
rs440446	chr19:44905910	C	G
-	chr19:44906337	G	A
rs769450	chr19:44907187	G	A
rs429358	chr19:44908684	T	C
rs7412	chr19:44908822	C	T
rs747519137	chr19:44909521	CT	C
-	chr19:44909967	TG	T
rs1065853	chr19:44909976	G	C,T
rs75627662	chr19:44910319	C	T
-	chr19:44910393	A	C
-	chr19:44910397	T	C
-	chr19:44910405	A	C
-	chr19:44910410	A	C
rs72654469	chr19:44910678	T	C
rs72654473	chr19:44911142	C	A
rs439401	chr19:44911194	T	C
rs5828224	chr19:44911609	AT	A
rs445925	chr19:44912383	G	A,C
rs483082	chr19:44912921	G	T
rs59325138	chr19:44913034	C	T
rs584007	chr19:44913221	A	G
rs438811	chr19:44913484	C	T
rs390082	chr19:44913574	T	A,C,G
rs72654445	chr19:44913943	G	A
-	chr19:44914318	A	T
rs34954997	chr19:44914381	C	CTTCG

**Table 2:** Number of total and phased ground-truth variants. For each sample, the table shows the total number of homozygous and heterozygous variants identified by Illumina data and the number of heterozygous variants phased by 10× Genomics or trio analysis. SNVs = single nucleotide variants, Indels = insertion/deletion variants.

Sample Name	Homozygous Variants		Heterozygous Variants		Heterozygous Phased Variants	
	SNVs	Indels	SNVs	Indels	SNVs	Indels
NA12878	0	0	10	1	10	1
NA12892	0	0	4	0	4	0
V1	6	3	11	1	11	1
V2	4	0	6	2	6	1
V3	4	1	4	1	4	1

**Table 3:** ONT sequencing statistics. The total number of ONT reads and the number and percentage of PASS reads, along with the read length of each category.

Sample Name	Number of Reads	Reads Mean Length	Number of PASS Reads (%)	PASS Reads Mean Length (bp)	Number of PASS Reads Aligned	Number of PASS Reads Covering the Whole Region
NA12878	28,251	11,871	24,335 (86%)	12,046	24,330 (99.99%)	23,799 (97.82%)
NA12892	27,410	11,710	23,541 (86%)	12,046	23,541 (100%)	23,022 (97.80%)
V1	39,993	11,552	33,489 (84%)	12,042	33,486 (99.99%)	32,941 (98.37%)
V2	38,501	11,690	33,373 (87%)	12,044	33,371 (99.99%)	32,755 (98.15%)
V3	32,084	11,588	27,165 (85%)	12,046	27,162 (99.99%)	26,673 (98.20%)

**Table 4:** Number of variants identified by the ONT platform. The total number of correct and spurious variants identified in the samples based solely on ONT data. SNVs = single nucleotide variants, indels = insertion/deletion variants, TP = true positives (identified by ONT and Illumina), FN = false negatives (only Illumina), FP = false positives (only ONT).

	NA12878						NA12892						V1						V2						V3					
	SNVs			Indels			SNVs			Indels			SNVs			Indels			SNVs			Indels			SNVs			Indels		
Number of Reads	TP	FN	FP	TP	FN	FP	TP	FN	FP	TP	FN	FP	TP	FN	FP	TP	FN	FP	TP	FN	FP	TP	FN	FP	TP	FN	FP	TP	FN	FP
**10**	10	0	9	0	1	1	4	0	3	0	0	0	16	2	0	0	3	3	9	1	10	0	2	2	6	2	3	0	2	3
**30**	10	0	0	0	1	1	4	0	0	0	0	3	16	2	0	1	2	2	9	1	0	0	2	4	8	0	0	0	2	2
**60**	10	0	0	0	1	2	4	0	0	0	0	3	17	1	0	0	3	2	10	0	0	0	2	2	8	0	0	1	1	2
**100**	10	0	0	0	1	2	4	0	0	0	0	2	17	1	0	0	3	2	10	0	0	0	2	2	8	0	0	0	2	2
**300**	10	0	0	0	1	1	4	0	0	0	0	0	17	1	0	0	3	2	10	0	0	0	2	0	8	0	0	0	2	0
**600**	10	0	0	0	1	0	4	0	0	0	0	0	17	1	0	0	3	2	9	1	0	0	2	0	8	0	0	0	2	0
**1000**	10	0	0	0	1	0	4	0	0	0	0	0	16	2	0	0	3	1	8	2	0	0	2	0	8	0	0	0	2	1
**10000**	8	2	0	0	1	1	4	0	0	0	0	1	10	8	0	0	3	1	7	3	0	0	2	3	7	1	0	0	2	2

## Supplementary Materials

The following are available online at https://www.mdpi.com/1422-0067/21/23/9177/s1.
